# Regenerative medicine applications: An overview of clinical trials

**DOI:** 10.3389/fbioe.2022.942750

**Published:** 2022-11-25

**Authors:** Astgik Petrosyan, Paulo N. Martins, Kim Solez, Basak E. Uygun, Vijay S. Gorantla, Giuseppe Orlando

**Affiliations:** ^1^ GOFARR Laboratory for Organ Regenerative Research and Cell Therapeutics in Urology, Division of Urology, Children’s Hospital Los Angeles, Saban Research Institute, Los Angeles, CA, United States; ^2^ Department of Surgery, Transplant Division, UMass Memorial Medical Center, University of Massachusetts, Worcester, MA, United States; ^3^ Department of Laboratory Medicine and Pathology, University of Alberta, Edmonton, AB, Canada; ^4^ Massachusetts General Hospital, Shriners Hospitals for Children in Boston and Harvard Medical School, Boston, MA, United States; ^5^ Wake Forest Baptist Medical Center and Wake Forest Institute for Regenerative Medicine, Winston Salem, NC, United States

**Keywords:** regenerative medicine, stem cells, extracellular vesicles, COVID-19, tissue engineering, transplantation, bioengineering

## Abstract

Insights into the use of cellular therapeutics, extracellular vesicles (EVs), and tissue engineering strategies for regenerative medicine applications are continually emerging with a focus on personalized, patient-specific treatments. Multiple pre-clinical and clinical trials have demonstrated the strong potential of cellular therapies, such as stem cells, immune cells, and EVs, to modulate inflammatory immune responses and promote neoangiogenic regeneration in diseased organs, damaged grafts, and inflammatory diseases, including COVID-19. Over 5,000 registered clinical trials on ClinicalTrials.gov involve stem cell therapies across various organs such as lung, kidney, heart, and liver, among other applications. A vast majority of stem cell clinical trials have been focused on these therapies’ safety and effectiveness. Advances in our understanding of stem cell heterogeneity, dosage specificity, and *ex vivo* manipulation of stem cell activity have shed light on the potential benefits of cellular therapies and supported expansion into clinical indications such as optimizing organ preservation before transplantation. Standardization of manufacturing protocols of tissue-engineered grafts is a critical first step towards the ultimate goal of whole organ engineering. Although various challenges and uncertainties are present in applying cellular and tissue engineering therapies, these fields’ prospect remains promising for customized patient-specific treatments. Here we will review novel regenerative medicine applications involving cellular therapies, EVs, and tissue-engineered constructs currently investigated in the clinic to mitigate diseases and possible use of cellular therapeutics for solid organ transplantation. We will discuss how these strategies may help advance the therapeutic potential of regenerative and transplant medicine.

## Introduction

Regenerative medicine focuses on replenishing and repairing tissue or organs impaired by disease, trauma, or congenital issues. Cellular therapies, conditioned media, extracellular vesicles (EVs), and seeded cellular patches are promising therapeutic tools to combat various inflammatory conditions and diseases. A large body of pre-clinical research has shown that stem cell therapies can delay disease onset within multiple organs such as the kidney (Sedrakyan et al., 2012; Urt-Filho et al., 2016; Frank and Petrosyan, 2020), lung (Mei et al., 2007; Zhen et al., 2008, 2010; Garcia et al., 2013; Xu et al., 2018), heart (Wang et al., 2015; Galipeau et al., 2016; Miteva et al., 2017), and liver (Gilsanz et al., 2017; Tsuchiya et al., 2019) through immunomodulatory and paracrine mechanisms. Conditioned media and EVs derived from stem cells also demonstrate similar characteristics (Lener et al., 2015; Nassar et al., 2016; Bruno et al., 2017; Riazifar et al., 2017; Sedrakyan et al., 2017; Grange et al., 2019). Mesenchymal stromal cells (mesenchymal stem cells; MSCs), which are used mainly in clinical trials, have a potent self-renewal and differentiation capacity into multi-lineages and may be isolated from various adult tissues such as bone marrow (BM), adipose tissue, and fetal specimens (amniotic fluid and umbilical cord). Cellular therapies are also investigated for transplant medicine with the hopes of repairing marginal organs, minimizing ischemia-reperfusion injury (IRI), and inducing immune tolerance in solid organ transplantation (Leventhal et al., 2016; Sawitzki et al., 2020). In addition to stem cell therapies, immune cell therapies that specifically isolate and enrich anti-inflammatory immune cells are also investigated as a promising regenerative medicine tool towards treating inflammation, promoting tissue regeneration, and enhancing transplant tolerance (Zwang and Leventhal, 2017). Currently, clinicians and scientists have begun providing novel insights into optimizing cellular therapy in the clinical setting to provide a more deliverable, sustained, and impactful clinical benefit to patients (Okano and Sipp, 2020). However, further studies with larger patient cohorts are needed to show the efficacy of cellular therapies, conditioned media, extracellular vesicles (EVs), and seeded cellular patches for regenerative medicine. Here we will review results obtained from current clinical trials and novel cellular therapeutic options investigated towards clinical use. We will discuss how these findings and current novel techniques may help advance the potential therapeutic effects of cellular transplantations, EVs, and tissue-engineered constructs for regenerative medicine and transplantation.

### Cellular therapeutics

Promising pre-clinical research studies have shown the potential of multipotent mesenchymal stem cells (MSCs) transplantation as a regenerative medicine therapy option (Vu et al., 2014; Wang et al., 2021). Currently, the U.S. Food and Drug Administration (FDA) has approved a small set of therapies for clinical use (Table 1). Clinical trials have focused on using MSCs immunomodulatory, immunosuppressive, and regenerative potentials with hopes of treating chronic diseases and immune resetting of autoimmune disorders (Table 2). MSCs immunoregulatory properties are attributed to their secretion of numerous cytokines (anti-inflammatory factors: iNOS, IDO, PGE_2_, TSG6, HO1 and galectins, cytokines: TGFβ, IL-10, CCL2, IL-6 and IL-7, chemokines: IL-6, CXCR3, CCR5, CCL5, CXCL9-11) and putative angiogenic proteins (VEGF, PDGF, TGFβ) (Shi et al., 2018). The Mesenchymal and Tissue Stem Cell Committee of the International Society for Cellular Therapy (ISCT) has set standards to define ‘multipotent mesenchymal stromal cells’ (MSC) for both laboratory-based scientific investigations and pre-clinical studies (Dominici et al., 2006). Three guidelines must be met for the designation of MSC. Firstly, MSC must be plastic-adherent (tissue culture flasks) in cultured under standard conditions. Secondly, MSC (measured by flow cytometry) must have specific surface antigen (Ag) expression (95% expression of CD105, CD73 and CD90, with absence (5/2%) in expression of CD45, CD34, CD14 or CD11b, CD79a or CD19 and HLA class II). Thirdly, MSC must exhibit differentiation capabilities towards osteoblasts, adipocytes, and chondroblasts under standard *in vitro* differentiating conditions. Not all published clinical trials have adhered to these guidelines, limiting our ability to compare and contrast study outcomes and hindering the field’s progression (Table 2).

**TABLE 1:** A list of cellular and tissue engineered products with the proposed treatments currently FDA approved. All of the approved cellular products are hematopoietic progenitor cell derived from Cord Blood approved for disorders affecting the hematopoietic system. The tissue engineered scaffolds are allowed for the treatment of mucogingival conditions, cartilage defects of the knee, and thermal burns.

Product	Company	Treatment
ALLOCORD (hematopoietic progenitor cell, Cord Blood)	SSM Cardinal Glennon Children’s Medical Center	Disorders affecting the hematopoietic system that are inherited, acquired, or result from myeloablative treatment
CLEVECORD (hematopoietic progenitor cell, Cord Blood)	Cleveland Cord Blood Center	Disorders affecting the hematopoietic system that are inherited, acquired, or result from myeloablative treatment
Ducord (hematopoietic progenitor cell, Cord Blood)	Duke University School of Medicine	Disorders affecting the hematopoietic system that are inherited, acquired, or result from myeloablative treatment
GINTUIT (Allogeneic Cultured Keratinocytes and Fibroblasts in Bovine Collagen)	Organogenesis Incorporated	Allogeneic cellularized scaffold product indicated for topical (non-submerged) application to a surgically created vascular wound bed in the treatment of mucogingival conditions in adults
HEMACORD (Hematopoietic progenitor cell, cord blood)	New York Blood Center	Disorders affecting the hematopoietic system that are inherited, acquired, or result from myeloablative treatment
Hematopoietic progenitor cell, Cord Blood	Clinimmune Labs, University of Colorado Cord Blood Bank	Disorders affecting the hematopoietic system that are inherited, acquired, or result from myeloablative treatment
Hematopoietic progenitor cell, Cord Blood - MD Anderson Cord Blood Bank	MD Anderson Cord Blood Bank	Disorders affecting the hematopoietic system that are inherited, acquired, or result from myeloablative treatment
Hematopoietic progenitor cell, Cord Blood - LifeSouth	LifeSouth Community Blood Centers, Inc	Disorders affecting the hematopoietic system that are inherited, acquired, or result from myeloablative treatment
Hematopoietic progenitor cell, Cord Blood - Bloodworks	Bloodworks	Disorders affecting the hematopoietic system that are inherited, acquired, or result from myeloablative treatment
MACI (Autologous Cultured Chondrocytes on a Porcine Collagen Membrane)	Vericel Corp	For the repair of single or multiple symptomatic, full-thickness cartilage defects of the knee with or without bone involvement in adults
STRATAGRAFT	Stratatech Corporation	Treatment of adults with thermal burns containing intact dermal elements for which surgical intervention is clinically indicated (deep partial-thickness burns)

**TABLE 2:** A list of clinical trials using regenerative medicine applications. Each trial is identified by disease reference, patient gender, method of treatment, outcome, and International Society for Cellular Therapy Criteria Check (1, 2, 3). 1) MSC must be plastic-adherent when maintained in standard culture conditions. 2) MSC must express CD105, CD73 and CD90, and lack expression of CD45, CD34, CD14 or CD11b, CD79alpha or CD19 and HLA-DR surface molecules. 3) MSC must differentiate to osteoblasts, adipocytes and chondroblasts *in vitro*. Most clinical trials using MSC appeared to have the 1st and 2nd criteria mentioned, and a large difference was noted between the trials regarding cell number, type, from of transplantation, and culture conditions. Such variation allows for the identification of different forms of effect per experimental group but shows little consistency in the trials performed. Thus, it would be beneficial if clinical trials followed a clearer guideline with minor changes per experimental group to understand better the applicability and efficacy of cellular and tissue engineered therapies.

Diseases reference	Patients male/Female	Treatment	Outcomes	International society for cellular therapy criteria check (1,2, 3)
Atherosclerotic renovascular disease (Saad et al. (2017))	*n* = 14 (9/5)	Investigational new drug (IND) #15082 and intra-arterial injection of low dose (1.0 × 10^5^ cells/kg) or higher dose (2.5 × 10^5^ cells/kg) autologous adipose-derived MSCs	No side effects. Stimulated angiogenesis and modified immune function. Increased renal tissue oxygenation and cortical blood flow	1) Plastic-adherent, 2) Specific surface antigen (Ag) expression
Newly diagnosed Type 1 Diabetes (Ye et al. (2017))	*n* = 8 3/5	Insulin and intravenous injection of autologous hematopoietic stem cell conditioning with cyclophosphamide (200 mg/kg) and rabbit anti-thymocyte globulin (4.5 mg/kg)	Improved residual C-peptide secretion lowered anti-GAD titers and reduced exogenous insulin dosages. Decreased expansion and function of Th1 and Th17 cells	NA: Cells mobilized with cyclophosphamide (2.0 g/m2) and granulocyte colony stimulating factor (10 mg/kg/day), and then collected from peripheral blood by leukapheresis and cryopreserved
Systemic sclerosis (Zhang et al. (2017))	*n* = 8 3/5	Plasmapheresis and infusion of 1 × 10^6^ cells/kg allogeneic umbilical cord mesenchymal stem cells	Improved mean modified Rodnan skin score, lung function and computed tomography (CT). Decreased anti-Scl70 autoantibody titer and serum transforming growth factor-β and vascular endothelial growth factor	1) Plastic-adherent, 2) Specific surface antigen (Ag) expression, and 3) multipotent differentiation potential
Steroid-resistant acute graft versus host disease (Bloor et al. (2020))	*n* = 15 8/7	Intravenous infusion of CYP-001 (induced pluripotent stem cells-derived mesenchymal stromal cells) two dose 1 × 10^6^ to10^8^ cells/kg or 2 × 10^6^ to 2 × 10^8^ cells/kg	Safe and well tolerated, no adverse side effects	1) Plastic-adherent, 2) Specific surface antigen (Ag) expression, and 3) multipotent differentiation potential
Symptomatic ischemic heart failure (Bartunek et al. (2017))	*n* = 107 males	Endomyocardial infusion with a retention-enhanced catheter of 2.4 × 10^7^ bone marrow mesenchymal stem cells expanded and differentiated to cardiopoietic cells	Safe with neutral results. Future clinical trials should consider patient selection based on disease severity markers	1) Plastic-adherent, 2) Specific surface antigen (Ag) expression
Nonischemic Cardiomyopathy (Butler et al. (2017))	*n* = 22 males	Intravenous infusion of ischemia-tolerant human donor allogeneic bone marrow MSCs dosed at 1.5 ×10^6^ cells/kg	Safe, caused immunomodulatory effects, and was associated with improvements in health status and functional capacity	1) Plastic-adherent, 2) Some specific surface antigen (Ag) expression
Ischemic cardiomyopathy (Florea et al., 2017)	*n* = 30 27/3	Transendocardial injection of 2 × 10^7^ or 1 × 10^8^ allogeneic bone marrow-derived human mesenchymal stem cells	Both cell doses reduced scar size, only the 1 × 10^8^ dose increased ejection fraction. Optimal dose and delivery crucial for beneficial results	1) Plastic-adherent
Chronic kidney disease (Nassar et al. (2016))	*n* = 18 9/9	Intravenous and intra-renal arteries injection of 1 × 10^10^ p/g EVs derived from human cord blood mesenchymal stem cells	Safe and can ameliorate the inflammatory immune reaction and improve the overall kidney function in grade III-IV CKD patients	1) Plastic-adherent, 2) Some specific surface antigen (Ag) expression
Moderate to severe COVID-19 (Meng et al. (2020))	*n* = 9	Three cycles of intravenous infusion of 3 × 10^7^ allogeneic umbilical cord-derived mesenchymal stem cells (manufactured by Vcanbio Cell & Gene Engineering Ltd.)	Safe and well tolerated, reduced IL6	1) Plastic-adherent, 2) Specific surface antigen (Ag) expression, and 3) multipotent differentiation potential
Severe COVID-19 (Shu et al. (2020))	*n* = 12 8/4	Intravenous administration 2 × 10^6^ cells/kg allogeneic umbilical cord-derived mesenchymal stem cells (manufactured by The Jiangsu Cell Tech Medical Research Institute and The Jiangsu Cell Tech Biotechnology Co.)	No adverse reactions, C-reactive protein and IL-6 levels were significantly decreased, lymphocyte count returned to normal range and observed reduced lung inflammation	1) Plastic-adherent, 2) Specific surface antigen (Ag) expression
Severe COVID-19 (Shi et al. (2021))	*n* = 65	Infusion of three doses of 4 × 10^7^ umbilical cord-mesenchymal stem cells (by VCANBIO Cell & Gene Engineering Corp, Tianjin, China)	Safe and showed improvement in whole lung lesion volume	1) Plastic-adherent, 2) Specific surface antigen (Ag) expression, and 3) multipotent differentiation potential
COVID-19-induced acute respiratory distress syndrome (Hashemian et al. (2021))	*n* = 11 8/3	Three intravenous infusions 2 × 10^8^ cells umbilical cord MSCs (UC-MSCs; 6 cases) or placental MSCs (PL-MSCs; 5 cases)	Improved respiratory distress and reduce inflammatory biomarkers in some. Patients with sepsis or multiorgan failure poor candidates	1) Plastic-adherent, 2) Specific surface antigen (Ag) expression
COVID-19-induced pneumonia (Liang et al. (2020))	*n* = 1 female	Three intravenous infusions of 5 × 10^7^ umbilical cord mesenchymal stem cells with thymosin α1 and antibiotics daily injection	Safe and well tolerated, showed remission of inflammation symptom	1) Plastic-adherent, 2) Specific surface antigen (Ag) expression, and 3) multipotent differentiation potential
Severe COVID-19-induced pneumonia (Leng et al. (2020))	*n* = 7 4/3	Intravenous drip of 1 × 10^6^ cells/kg mesenchymal stem cells suspended in 100 ml of saline	Safe and well tolerated, reduced inflammatory response, promoted tissue repair and regeneration	NA: No information classified as clinical grade MSCs
Severe COVID-19 (Sengupta et al. (2020))	*n* = 27 17/10	Intravenous drip of 15 ml of exosomes (ExoFlo™) derived from allogeneic bone marrow mesenchymal stem cells	Safe and well tolerated, restored oxygenation, downregulated cytokine storm, and reconstituted immunity	NA
COVID-19 pulmonary fibrosis (Wu et al. (2020))	*n* = 27 19/8	1 or 2 or 3 intravenous transfusion of 3 × 10^6^ cells/kg embryonic stem cell–derived immunity- and matrix-regulatory cells	Safe and well tolerated, with improved clinical symptoms and reduced pulmonary fibrosis	NA
Nonacute stroke ischemic or hemorrhagic (Chernykh et al. (2016))	*n* = 13 12/1	Intrathecally injection of 2.19 × 10^7^ macrophage type 2 generated from autologous peripheral blood mononuclear cells in 2 ml of saline	Safe and improved neurological recovery possibly by immunomodulatory activity	NA
Living donor kidney transplant recipients (Mathew et al. (2018))	*n* = 9 6/3	Infusion of 0.5, 1, or 5 × 10^9^ CD4^+^CD25^+^ Tregs isolated from patient’s cryopreserved leukopheresis and expanded *in vitro*	Safe and showed no adverse infusion related side effects, infections or rejection events up to 2 years post-transplant	NA
Urethral stricture recurrences urethroplasty (Ram-Liebig et al. (2017))	*n* = 99 male	Tissue-engineered oral mucosa graft generated from oral mucosa biopsy and manufactured by MukoCell®	Safe and efficient in urethroplasty	NA
Chronic nonhealing venous leg ulcers (Stone et al. (2017))	*n* = 15 13/2	FDA-approved bilayered living cell construct consists of human foreskin-derived neonatal fibroblasts in a bovine type I collagen matrix below a layer of human foreskin-derived neonatal epidermal keratinocytes	Safe and well tolerated, changed inflammation to acute healing process	NA
Chronic myocardial scar (Bayes-Genis et al. (2016))	*n* = 5	Autologous pericardial adipose graft (adipose tissue taken from the left or the right side of the pericardium)	Safe procedure that may be efficacious in selected patients	NA
Severe ischemic left ventricular dysfunction (Menasche et al. (2018))	*n* = 6 5/1	Embryonic stem cell differentiated toward cardiovascular lineage than mixed with fibrinogen and thrombin to form a gel	Safe and showed low risk in short- and medium-term adverse events	NA
Chronic non-ischemic dilated cardiomyopathy (Hare et al. (2017))	*n* = 34 24/10	Transendocardial injection in ten left ventricular sites by NOGA Catheter of 1 × 10^8^ allogeneic or autologous bone marrow-derived mesenchymal stem cells	Allogeneic MSCs safe and efficacious alternative to autologous MSCs. Therapeutic effects driven by immunomodulation and endothelial restoration	1) Plastic-adherent
Type 2 Diabetes Mellitus (Bhansali et al. (2017))	*n* = 20 15/5	Superior pancreatico-duodenal artery injection of 1 × 10^6^ cells/kg of *in vitro* expanded autologous bone marrow-derived mesenchymal stem cells or 1 × 10^9^ autologous bone marrow- derived mononuclear cells (separated by centrifugation)	Both cell types resulted in sustained reduction in insulin doses. MSC showed better improvement in insulin sensitivity while MNC showed an increase in C-peptide response	1) Plastic-adherent, 2) Specific surface antigen (Ag) expression
Dilated cardiomyopathy (Xiao et al. (2017))	*n* = 33 21/12	Intracoronary administration of (5.1 ± 2.0) × 10^8^ bone marrow mononuclear cells or (4.9 ± 1.7) × 10^8^ mesenchymal stem cells	Safe and both show comparable effectiveness. BMSC showed further improvements at 12-month but not BMMC.	1) Plastic-adherent, 2) Specific surface antigen (Ag) expression
Autosomal dominant polycystic kidney disease (Makhlough et al. (2017))	*n* = 6 3/3	Infusion through the cubital vein of 2 × 10^6^ cells/kg autologous cultured bone marrow mesenchymal stromal cells	Safe and well tolerated	1) Plastic-adherent, 2) Specific surface antigen (Ag) expression
Alcoholic hepatitis (Lanthier et al. (2017))	*n* = 28 14/14	Hepatic artery infusion of 0.47 ± 0.15 × 10^8^ cells/kg bone marrow derived CD34^+^ stem cells and mesenchymal stem cells	No clinical efficacy detected	NA: cells mobilized with 5-day course of lenograstim and centrifuged over a Ficoll-Hypaque plus gradient
End-stage liver disease (Rajaram et al. (2017))	*n* = 1 male	Two intrahepatic arterial infusion of 1.2 × 10^6^ autologous bone marrow derived mesenchymal stem cells expanded *in vitro*	Safe with only short-term clinical benefit	1) Plastic-adherent, 2) Some specific surface antigen (Ag) expression
Decompensated liver cirrhosis (Kim et al. (2017))	*n* = 19 9/10	Peripheral vein infusion of 1 × 10^8^/kg bone marrow mononuclear cells	Short term improvement of liver function and volume. High incidence of hepatocellular carcinoma	NA

In current clinical trials, similar to pre-clinical data, clinical administration of cellular therapies has shown angiogenic properties (active secretion of proangiogenic factors) and anti-inflammatory effects (reduced expression of pro-inflammatory markers and T cell proliferation) (Saad et al., 2017; Ye et al., 2017; Zhang et al., 2017). The angiogenic properties of autologous adipose tissue-derived MSCs are attributed to significantly increasing renal tissue oxygenation, cortical blood flow, and stabilizing glomerular filtration rates (GFR) up to 3 months in patients with the atherosclerotic renovascular disease (RVD) (Saad et al., 2017). The anti-inflammatory effects of autologous hematopoietic stem cells are predicted to be beneficial for patients with type 1 diabetes mellitus by lowering the proportion of white blood cells, lymphocytes, T-cell proliferation, and pro-inflammatory cytokine production (Ye et al., 2017). Similarly, anti-inflammatory properties of allogeneic umbilical cord-derived MSCs, show improvements in patients with systemic sclerosis-associated, with better skin thickness scores, lung function, significantly decrease in anti-Scl70 autoantibody titers, and reduction of pro-inflammatory cytokine levels (including transforming growth factor-β (TGF-β) and vascular endothelial growth factor (VEGF) levels in serum) (Zhang et al., 2017). Although clinical trials show promising results for MSC use in the clinic, there are limitations in MSCs scalability, interdonor variability, clinical trial outcomes inconsistency, low engraftment rates, variation in immunomodulatory response, and potential regenerative limitations (Tanavde et al., 2015). Recently, induced pluripotent stem cells (iPSCs) derived MSCs (CPY-001) are shown to be safe and well-tolerated in a limited number of patients with steroid-resistant acute graft versus host disease (Bloor et al., 2020). This trial demonstrates for the first time, the possible applicability of iPSC-derived MSCs for a range of other clinical targets that may overcome the fundamental limitations of conventional, donor-derived MSC production processes. Although current clinical trials exhibit similar and limited anti-inflammatory beneficial effects with MSC treatments like previous pre-clinical trials, there is a large variation between each trial. Variations such as cell culture conditions, cell number transplantation, from of transplantation, cell type, and characterization limited the interpretation of each trial. Additional studies with larger cohorts are also needed to address the efficacy of cellular therapeutics in regenerative medicine.

### Optimization of cellular therapeutics through modification of dosing, timing, route, and frequency of administration and activation of endogenous cells

MSCs preconditioned with either recombinant proteins, drugs, or *ex-vivo* cell culture conditions and techniques are also investigated to enhance their therapeutic potential before transplantation (Table 2). One form of enhancement strategy applied for cardiac regenerative cell therapy is using a guided cardiopoiesis approach to deliver BM-MSCs expanded and processed for lineage specification to derive cardiopoietic cells. In a Phase III Congestive Heart Failure Cardiopoietic Regenerative Therapy (CHART-1) clinical trial, cardiopoietic stem cells were delivered *via* the endomyocardial route with a retention-enhanced catheter to patients with ischemic heart failure (Bartunek et al., 2017). However, after thirty-nine weeks, the primary outcome was neutral, except for a subset of patients with severe heart enlargement that appeared to have had a consistent beneficial effect. The results suggest that cardiopoietic cell treatment beneficial outcomes may vary depending on the type of cardiac damage present in patients. Another alternative method used to enhance MSC regenerative potential aside from preconditioning the cells with recombinant growth factors, cytokines, or drugs is the use of environmental stimuli, such as hypoxia. Preconditioned MSCs under chronic hypoxic conditions (itMSC) show enhanced immunomodulatory properties when transplanted in non-ischemic cardiomyopathy patients (Butler et al., 2017). After 90 days, the administration of itMSCs was associated with a reduced number of natural killer cells, and the magnitude of this reduction was correlated with improved left ventricular ejection fraction (Butler et al., 2017). However, a single injection of itMSC was not efficient in promoting significant cardiac structural or functional improvements, highlighting the need to investigate the efficacy of serial dosing of intravenously administered itMSCs to promote a sustained immunomodulatory effect along with structural and functional improvements in the clinic. Thus, clinicians have also carried out studies identifying how different dosages of stem cells and numbers of injections (transplants) may dictate their therapeutic potential. In the TRIDENT Study, Florea et al. have demonstrated in patients with ischemic cardiomyopathy that there are different beneficial outcomes when patients are administered either 20 million or 100 million allogeneic MSC *via* transendocardial injection (Florea et al., 2017). Both groups showed improvement in scar formation; however, improved ejection fraction was noted only in patients receiving 100 million cells. The authors stated that although the two doses of allogeneic MSC are safe for patients, it is crucial to design trials to evaluate optimal dosing for cell-based therapies. Clinical trials have also begun to understand how different cell types produce better results than single-cell transplantation. In pre-clinical studies (Park K.-S. et al., 2019), have recently demonstrated that delivering both cardiomyocytes derived from human induced pluripotent stem cells (hiPSC-CMs) and human mesenchymal stem cell-loaded patch (hMSC-PA) to rats with myocardial infractions can amplify cardiac repair with enhanced vascular regeneration and improved cellular retention and engraftment (Park S.-J. et al., 2019). The combinatory cell delivery can also be applied to organ transplantations to enhance/preserve newly transplanted partial organ engraftment. Benomar et al. demonstrated that patients who were transplanted with pancreatic tissue comprising more than 50% of non-islet cells (likely enriched in ductal, acinar, and MSCs) had a statistically significant lower level of hemoglobin A1c and lower daily requirement of insulin even 5 years after transplantation, compared to those who received islet transplant with more than 50% tissue purity (Benomar et al., 2018); thus, suggesting that non-endocrine cells have a beneficial effect on long-term islet graft metabolic function. The authors identified elevated expression of CA19-9 generally synthesized by pancreatic ductal cells and hypothesized that ductal cells must have been transplanted and continued to proliferate and contributed to the beneficial outcomes. These findings bring forth an important concept, the need to transplant multiple cell types for better long-term engraftment and function. These results suggest and warrant further investigation into the understanding and application of methods to enhance the therapeutic potential of MSC, either through improved cell culture techniques, the route of delivery, dosage specificity, or a combination of various cell types to further amplify their regenerative potential.

Current therapies are also designed to mobilize patients’ tissue-specific progenitor cells using various bioactive molecules such as growth factors, cytokines, and hormones to enhance endogenous regeneration. Activation of endogenous stem cells to promote regeneration or repair holds great promise for the future of translational medicine (Xia et al., 2018). Ansheles et al. demonstrated that using statins (Atorvastatin therapy) in patients with coronary heart disease could significantly increase the pool of endothelial progenitor cells by 72% in 3 months (Ansheles et al., 2017). Patients also displayed a significant decrease in VEGF expression and various metabolic markers such as C-reactive protein, total cholesterol, LDL cholesterol, and triglycerides. Pantin et al. also investigated how to enhance endothelial cell mobilization from patients following allogeneic transplantation to sustain donor-derived hematopoiesis (Pantin et al., 2017). They identified that a high-dose (480 mg/kg) Plerixafor is safe and effective in mobilizing CD31 expressing cells in healthy donors. These studies highlight the use of molecules to enhance tissue regeneration and restoration in disease by activating endogenous resident cells without the need for exogenous cellular infusions.

### Extracellular vesicle therapeutics

Cell-to-cell communication is vital to control wound healing and modulate chronic and acute diseases *via* paracrine signaling. Cells communicate *via* the secretion of numerous extracellular vesicles (EVs) which are a heterogeneous population and ranging from 40 nm to a few mm in size under physiological and pathophysiological conditions. EV populations most widely studied and characterized are exosomes (derived from intracellular endosomal compartments and range from 30 to 120 nm in diameter), microvesicles (also known as shedding vesicles are non-apoptotic EVs that originate from the plasma membrane and range from 50 to 1,000 nm in diameter), and apoptotic bodies (originate from cells undergoing apoptosis and range from 50 to 2,000 nm). Multiple pre-clinical studies have demonstrated that conditioned media of cultured stem cells and stem cell EVs show beneficial effects on various diseases (Lener et al., 2015; Bruno et al., 2017; Riazifar et al., 2017; Nguyen et al., 2020). The discovery of exosomes, microvesicles, and apoptotic bodies within the conditioned media has led to a new avenue of research exploring EVs for clinical use. Using EVs, most of the therapeutic effects of stem cells can be achieved with a reduced risk associated with live-cell injection late effects, such as neoplastic transformation and immune response activation (Nassar et al., 2016; Wang et al., 2017; Guo et al., 2020). A limited number of clinical trials have investigated EVs’ therapeutic potential in patients with cancer (Morse et al., 2005; Dai et al., 2008) and disease (Nassar et al., 2016). In chronic kidney disease patients (Table 2), EVs isolated from umbilical cord MSCs were shown to be safe and potentially effective in modulating the inflammatory immune reaction (Nassar et al., 2016). Patients who were given two doses of MSC-EVs showed improved eGFR, serum creatinine level, blood urea, and urinary albumin-creatinine ratio, possibly due to a significant plasma level increase in TGF-β1 and IL-10 with a decrease in plasma levels of inflammatory cytokine, TNF-α. Although patients saw a vast improvement after two dosages of therapy at 8 weeks to 9 months, the improvements were not sustained after 9 months, and an additional administration of the EV might be needed (Nassar et al., 2016).

Further studies are also necessary to clarify fundamental questions regarding the generation, origin of isolation (body fluids: plasma, serum, blood, amniotic fluid, cell lines: MSCs, progenitor cells, IPSC’s distribution, tissue derived) (Crescitelli et al., 2021) and uptake of EVs and how to scale up to cGMP manufacturing and improve associated quality control and batch tracking methods for the clinic (Riazifar et al., 2017). Another issue brought forth by the International Society for Extracellular Vesicles is the general lack of proper characterization of the different forms of EVs used in pre-clinical and clinical trials as each type contains different cargos and may promote different effects (Théry et al., 2018). There are currently multiple clinical trials initiated and recruiting patients to investigate EVs’ application in various diseased organs such as lung, liver, kidney, and heart. The potential use of EVs as a regenerative medicine therapeutic option is vast and promising. There are currently no FDA-approved EV products.

### Cellular therapeutics to improve donor organ quality

Cellular therapeutics have also been applied *ex vivo* to improve and recondition donor organ quality before transplantations. Thompson et al. show how *ex vivo* delivery of multipotent adult progenitor cells *via* normothermic machine perfusion in kidneys deemed un-transplantable prompted improved clinically relevant parameters (urine output, decreased expression of injury biomarker NGAL, improved microvascular perfusion) and decreased neutrophil recruitment and pro-inflammatory cytokines (downregulation of interleukin (IL)-1β, upregulation of IL-10 and Indolamine-2, 3-dioxygenase) (Thompson et al., 2020). Brasile et al. also show how 24 h *ex vivo* perfusion of MSC in an Exsanguinous Metabolic Support tissue-engineering can accelerate the repair of ischemic damage in human kidneys. Promoting regeneration identified by the increased synthesis of ATP (both in the renal cortex and medulla), a reduced inflammatory response (TNF-α, RANTES, IL1-B, IL6), increased synthesis of growth factors (EGF, FGF-2, and TGF-α), normalization of the cytoskeleton (ZO-1 expressed exclusively at the plasma membrane) and increased cellular proliferation (higher expression of PCNA and mitosis) (Brasile et al., 2019). The authors suggest a more prolonged warm reperfusion of a donor’s kidney may further improve and repair tubule damages attained from severe ischemic insult. The potential of MSCs to prevent or decrease injuries due to ischemia-reperfusion to further improve organ preservation has also been shown in various organs such as the lung (La Francesca et al., 2014; Lu et al., 2015), liver (Laing et al., 2020), and heart (Yano et al., 2018). Thus, these techniques involving reperfusion using various cell types provide a new avenue to significantly expanding donor criteria to offset current donor shortages. Future studies directed towards identifying the precise reperfusion media, the extent of reperfusion time, and the most suitable cell source can further enhance these techniques’ applicability in the clinic.

### COVID-19 therapies

COVID-19, the disease attributed to the novel SARS-CoV-2 coronavirus, has given rise to a global pandemic. Although many patients do well, some present fever, dyspnea, hypoxia, and even exhibit moderate-to-severe acute respiratory distress syndrome (ARDS). This group of patients typically require intubation, which is associated with high mortality rates (up to 67%–94%) (King et al., 2020). The detrimental effect of COVID-19 that causes multiple organ failure and even death is correlated with the presentation of a cytokine storm, which is identified as a maladaptive release of cytokines (Brodin, 2021). Elevated expression of inflammatory cytokines such as IL-1B, IFN-γ, IP-10, and monocyte chemoattractant protein 1 (MCP-1) detected in patients with COVID-19 is linked with Th1 cell response (Ye et al., 2020). Currently, MSC and their EVs are considered as a potential therapeutic option against COVID-19 (Table 2). MSC has the innate capacity to promote anti-inflammatory and immune regulatory functions by directly inhibiting abnormal activation of T lymphocytes and macrophages, pro-inflammatory cytokines, and secreting anti-inflammatory cytokines and growth factors such as IL-10 and VEGF to stimulate regeneration and repair. There are currently 16 clinical trials completed with over one thousand studies listed on ClinicalTrials.gov on the use of stem cells or stem cell exosomes to treat coronavirus-related injuries, such as acute kidney and lung injury and various inflammatory processes. Non-randomized case studies, phase 1 and phase 2 clinical trials have shown that human umbilical cord-derived mesenchymal stem cell (UC-MSCs) infusions in patients with moderate and severe COVID-19 pulmonary disease is safe and well-tolerated (Liang et al., 2020; Meng et al., 2020; Shu et al., 2020; Hashemian et al., 2021; Shi et al., 2021). A phase 1 and phase 2 clinical trial with limited patients shows that administration of UC-MSCs or clinical-grade MSCs may help reduce inflammatory cytokines (TNF-α, IFN-γ, IL6, IL8, C-reactive protein) and promote lung recovery in surviving patients (Liang et al., 2020; Hashemian et al., 2021; Shi et al., 2021). Intravenous injection of clinical-grade MSCs (lacking ACE-2 receptor and TMPRSS2) led to increased levels of anti-inflammatory cytokine IL-10, and the normalized presence of immune cells. The patients presented an increase of peripheral lymphocytes, a decrease in C-reactive protein (CRP), a reduced activated cytokine-secreting immune cells (CXCR3+CD4+T-cells, CXCR3+CD8+Tcells, and CXCR3+NK-cells), and a restored levels of regulatory DC cell population (CD14^+^CD11c+CD11bmodregulatory DC cell) (Leng et al., 2020). The use of MSC with the absence of ACE-2 receptor and TMPRSS2 to prevent infection with SARS-Cov-2 may have enhanced the therapeutic effects of MSCs.

There are currently multiple studies listed on ClinicalTrials.gov on the use of EVs to treat COVID-19. Sengupta et al. show that a single dose of intravenous infusion of exosomes derived from BM-MSC (ExoFloTM) in patients presenting moderate-to-severe ARDS helps restore oxygenation, reduces the cytokine storm, to bring back a healthy immune system with no adverse effects (Sengupta et al., 2020). The authors state that exosomes may be used as a preventative measure against progression to invasive oxygen support and mechanical ventilation, which is associated with a high mortality rate. Further studies with randomized controlled trials (RCTs) are warranted to prove efficacy and address what type of EVs and what dosage of EVs are needed to treat COVID-19 patients. A short-term (84 days) Phase 1 clinical trial of twenty-seven COVID-19 patients with pulmonary fibrosis treated with human embryonic stem cell-derived immunity and matrix-regulatory cells, which poses high expression of proliferative, immunomodulatory and anti-fibrotic genes, also show improvements in clinical symptoms (Wu et al., 2020). Additional multicenter randomized placebo-controlled Phase 2/3 trials are underway for further proof. Although these findings are promising, additional studies with larger cohorts are needed to assess the efficacy of MSCs and EVs therapeutic potential to treat and prevent the progression of COVID-19 related injuries in patients. While many clinical trials are listed, not all have begun, and only a few have been completed. Additionally, the completed trials consist of a small sample size, various cellular products, different culture methodology, and need more time for result interpretation. Leading to a discouraging notion that COVID-19 treatment with cellular therapies may not be available soon to treat a significant number of patients. COVID-19 clinical trial moving forward should focus on clear identification of cellular products used and improve quality of study design to further the future of cellular therapies in treatment of COVID-19.

### Immune cellular therapies

Aside from using stem cells, the field of regenerative medicine also investigates the potential isolation and enrichment of specific anti-inflammatory immune cells to treat inflammation, promote tissue regeneration and transplant tolerance (Table 2). In non-acute stroke patients, administration of autologous M2 macrophages is shown to be safe and can modulate inflammatory responses, contributing to angiogenesis and tissue repair (Chernykh et al., 2016). However, the treatment appeared to be more effective in patients with lower endogenous immunosuppressive mechanisms (IL-10, FGF-β, PDGF, VEGF) and increased pro-inflammatory activity (IL-1β, TNF-α, IFN-γ, IL-6). Infusion of autologous Treg cells has also been investigated for kidney transplantation patients to promote transplant tolerance in hopes of avoiding long-term use of toxic immunosuppressive agents that cause increased morbidity/mortality (Mathew et al., 2018). The administration of transplanted polyclonal Tregs (CD4^+^CD25^+^ T cells) derived from the thymus or peripheral tissues of the recipients and expanded *in vitro* into living donor kidney transplant recipients showed a reduction of total CD4+T and CD8^+^ T cells and a 5–20 fold increased circulating Tregs levels after 90 days. The authors aim to move into a phase II clinical trial to test Treg infusion’s efficacy for tolerance induction or drug minimization (Mathew et al., 2018). Chimeric antigen receptor transduced natural killer (CAR-NK) therapy (Liu et al., 2020), and pluripotent stem cell-derived immunosuppressive cells (macrophages) (Tsuji et al., 2020) are also investigated for use in solid organ transplantation as an alternative method of posttransplant management to improve allograft survival and minimize secondary complications. Recently, Tsuji et al. showed the successful generation of immunosuppressive cells from non-human primate ESCs that expressed several immunosuppressive molecules and significantly inhibited allogeneic mixed lymphocyte reaction (Tsuji et al., 2020). The future goal is to move into pre-clinical trials and demonstrate their potential to suppress allogeneic immune reactions against grafts derived from the same donor in transplantation models. Although advancements in surgical technique and immunosuppression regimens have progressed in transplant medicine, many limitations still exist. The chronic use of immunosuppression in transplant medicine promotes several side effects and increases the relative risk of infections, malignancy, cardiovascular morbidity, and organ damage (e.g., liver toxicity, nephrotoxicity, neurotoxicity, and diabetes mellitus). Thus, to further improve solid organ transplantation outcomes, discovering a novel immunoregulating strategy in regenerative medicine using pluripotent stem cells and engineered immune cells to enhance organ survival and tolerance is vital for the growth of transplant medicine.

### Tissue engineered grafts

In tissue engineering, a combination of cells, a scaffold, and biologically active molecules are used to reconstruct or regenerate damaged tissues or whole organs. The success of tissue engineering relies on the interplay between multiple scientific disciplines such as cell biology, biomedical engineering, and material science. The identification of proper scaffolds, bioreactors, cell sources, and biomolecules such as growth factors and chemokines are needed to reconstruct or regenerate organs correctly. Currently, contrary to 2D planar tissues, bioengineering solid organs for transplantation is still challenging. Advances have been made towards identifying novel scaffolds, biomolecules, and cells, but protocols towards combining the mixture for solid organs’ *de novo* reconstruction are still a limiting factor. Although scientific thinking and approaches towards fully realizing the exciting potential of whole organ engineering are still in their early phases, there have been advances in using novel technology with cell therapy to enhance tissue regeneration and function in the clinic (Table 2).

Tissue engineering is currently applied to creating alternative materials for the reconstruction of multiple organs. Ram-Liebig et al. show that manufactured tissue-engineered oral mucosa graft is safe and efficient in urethroplasty in male patients with surgically unsuccessful pretreated urethral stricture (Ram-Liebig et al., 2017). The procedure involves harvesting a small oral biopsy from the patients and sending it out to a Good Manufacturing Practice (GMP) laboratory manufacturing company, MukoCell®, where the sample is used to create a tissue-engineered oral mucosa graft for the urethroplasty. The transplant success rate was 67.3% at 12 and 58.2% at 24 months and the authors hypothesize that the success rate may be higher if the patients are initially treated with the graft from the beginning. Nonetheless, the authors show that the bulbar and penile urethra reconstruction is feasible, safe, and efficacious in a heavily pretreated population using a tissue-engineered oral mucosa graft. This study demonstrates how current tissue engineering therapies could be successfully standardized and manufactured in a company to provide a constant viable product tailored to everyone.

Clinical studies are also exploring the mechanisms of how tissue-engineered constructs cross-communicate with the diseased milieu to promote healing of a chronic wound. Stone et al. used transcriptomics to understand mechanistically how an FDA-approved bilayer living cell construct (BLCC) promotes the healing of chronic non-healing venous leg ulcers (Stone et al., 2017). BLCC consists of a layer of the human foreskin–derived neonatal fibroblasts in a bovine type I collagen matrix under a layer of the human foreskin–derived neonatal epidermal keratinocytes. The authors identified that BLCC provides bioactive signals after transplant to the damaged tissue site to promote wound healing *via* modulation of inflammatory and growth factor signaling, keratinocyte activation, and attenuation Wnt/β-catenin signaling. This study identifies mechanistically how tissue-engineered constructs can communicate at the site of injury to promote healing (Stone et al., 2017). The use of a cardiac patch has also garnered much attention, which provides cells a proper microenvironment for tissue development and maturation (Menasché et al., 2018). Bayes-Genis et al. have shown that autologous pericardial adipose graft transplanted within patients treated with coronary artery bypass graft surgery promotes a noticeable improvement in reducing the necrotic mass-sized ventricular volumes after 1 year (Bayes-Genis et al., 2016). The authors used an autologous pericardial adipose graft directly obtained from the patients and surgical glued it in place over the necrotic zone after the coronary artery bypass. The surgeons harnessed the biological regenerative capacity of adipose tissue for patients with a chronic myocardial scar. However, no statistically significant difference was noted in necrosis size, possibly due to the limited patient numbers and the need to refine the surgical procedure (Bayes-Genis et al., 2016). Cardiac patches are also used to address the limitation in the retention and need of large cell numbers for cardiac regenerative therapy. In a phase I clinical trial, Menasche et al. assessed the safety and efficacy of transplanting human embryonic stem cell (hESC)-derived cardiovascular progenitors embedded in a fibrin patch in severe ischemic left ventricular dysfunction patients receiving a coronary artery bypass procedure (Menasché et al., 2018). The cardiac fibrin patch showed no evidence of tumor formation or arrhythmias during the 18 months follow-up. Although the feasibility of producing clinical-grade hESC-CM for transplantation was demonstrated, clinical trials assessing efficacy were not yet conducted due to the small sample size, lack of blinded assessment, and confounding effect of the associated coronary artery bypass grafting. Based on these results, there is still a need to identify the best source of stem or progenitor cells and extracellular matrix or biomaterial to promote tissue regeneration and repair in efficacy and safe manner.

### Challenges and hurdles of cellular therapies

Researchers have identified how stem cell heterogeneity, due to differences in source and donor to donor variations, may limit their clinical effectiveness. Autologous (isolated from and transplanted back into the same patient) and allogeneic (isolated from a different patient) stem cells have a different beneficial therapeutic potential based on disease and organ model. Hare et al. demonstrate that although transplantation of both autologous and allogeneic BM-MSCs is safe, feasible, and beneficial when applied in chronic non-ischemic dilated cardiomyopathy (NIDCM), there are slight differences in their beneficial outcomes (Hare et al., 2017). Allogeneic BM-MSCs transplants promote a more significant improvement in functional tests like Ejection Fraction (EF), Minnesota Living with Heart Failure Questionnaire (MLHFQ), Six Minute Walk Test (6MWT), along with the better functional restoration of endothelium and reduction of pro-inflammatory cytokines (TNF-α) 6 months after transplantation compared to autologous BM-MSCs. Similarly, Bhansali et al. also show that autologous bone-marrow or mononuclear cells (MNCs) transplanted in patients with type 2 diabetes mellitus effectively reduce the need for insulin after a year (Bhansali et al., 2017). However, patients with MNC transplants showed a significant increase in second-phase C-peptide response during the hyperglycemic clamp indicating insulin production, while MSC transplanted patients had a significant improvement in insulin sensitivity index and an increase in insulin receptor substrate-1 gene expression. Thus, demonstrating the need for more informative studies to distinguish the differential beneficial effects of different cell cellular therapies. Xiao et al. also compared the efficacy of intracoronary administration of BM-MNCs or BM-MSCs for patients with dilated cardiomyopathy (DCM) (Xiao et al., 2017). After 3 months, both injections showed an improvement in New York Heart Association (NYHA) functional class and left ventricular ejection fraction (LVEF) in patients. However, after 12 months, BM-MSCs transplanted patients continued to significantly improve LVEF and NYHA, unlike BM-MNCs transplanted patients who showed a decrease in LVEF compared to their 3 months follow-up. These results suggest that BM-MNCs provided a temporary improvement in LVEF and NYHA class and only accelerate cardiac function recovery while the improvement observed following BM-MSC therapy is sustained (Xiao et al., 2017). These studies provide novel insights and a comprehensive understanding of how various cell sources and cell types may deliver different therapeutic effects based on disease. Additionally, they highlight the need to tailor stem cell therapies specific to each patient’s need to enhance their regenerative potential. Further conformational studies with large, randomized, placebo-controlled clinical trials are needed to clarify the complexity of MSCs (based on origin and application) and their interaction with host tissue.

Although advancements are being made daily in cellular therapy, there are still many challenges in translating pre-clinical results regarding cellular therapy efficacy to promote tissue healing, reduce excessive inflammation, and improve the clinic’s survival (Galipeau et al., 2016; Chinnadurai et al., 2018). It has been shown that not all stem cell therapies are initially beneficial. Makhlough et al. show the safety and tolerability of autologous BM-MSC transplanted into six autosomal dominant polycystic kidney disease patients but with no physiological improvement detected after 1 year (Makhlough et al., 2017). Patients exhibited a continuous decrease of GFR with a significant increase in serum creatinine levels. The study was limited to only six patients, and only a single cell transplant was administered, which may partially explain the limited beneficial effects detected (Makhlough et al., 2017). Stem cell therapy’s effectiveness may also be limited by the extent of chronic inflammation and fibrosis already present within the patient’s damaged tissue. In patients with decompensated (severe) alcoholic liver disease, transplantation of BM-MSCs showed no modification of the disease’s progression after 4 weeks (Lanthier et al., 2017) to 8 weeks (Rajaram et al., 2017). Although patients showed an elevation of liver macrophages and upregulation of regenerative liver markers (SPINK1 and HGF), no difference was detected regarding proliferative hepatocyte numbers (Lanthier et al., 2017). There are also potential safety concerns with cellular therapy, such as the potential for malignant transformation of MSCs (Steinemann et al., 2013). A long-term follow-up study of patients with decompensated (severe) alcoholic liver disease transplanted with autologous BM-derived mononuclear cells showed improved liver function and decreased collagen levels in patients’ liver transiently 6 months post-transplantation (Kim et al., 2017). Patients also displayed improved biochemical parameters, CP class, and increased liver volume, indicating liver regeneration. Although improved liver function was still evident at the five-year follow-up, patients who had received cell transplantation had an alarming increased risk of developing hepatocellular carcinoma (HCC). This relatively high incidence of HCC within 2 years after autologous bone marrow cell infusion warrants further investigation (Kim et al., 2017). Other studies have also shown that a small group of hematopoietic cell transplant survivors may suffer from not only solid tumors but also from other significant late effects such as diseases of the cardiovascular, pulmonary, and endocrine systems, dysfunction of the thyroid gland, gonads, liver and kidneys, infertility, iron overload, bone diseases, infection, and neuropsychological effects (Inamoto and Lee, 2017). The leading cause of mortality in adult patients who had received hematopoietic cell transplants includes recurrent malignancy, lung diseases, infection, secondary cancers, and chronic graft-versus-host disease. Thus, long-term risk assessment studies of patients receiving stem cell transplantation are needed to understand the risk of developing cancer and other harmful late effects versus the long-term benefits of stem cell therapy. Another limitation preventing comparison of current clinical trials’ and their outcomes, is that not all clinical trials adhered to ISCT criteria in defining the cellular treatments. Moving forward, improved methodological quality, increased sample size, and extended trial duration are needed for a better comparison of clinical trial data and results amongst each study. Cossu et al. and others also emphasized the need for better science, funding models, governance, public and patient engagement to enhance cellular therapy’s efficacy and safety in the clinic (Cossu et al., 2018). Regulatory limitations are another hurdle for the application of cell therapies or new technologies. With growing innovations made in regenerative medicine, outdated regulations may not adequately address new challenges posed as technology advances. Thus, new regulations must be designed to protect the patients from unnecessary risk while encouraging investigators, funding bodies, and investors to support research and development and market commercialization of novel products.

## Conclusion and future directions

**FIGURE 1:**
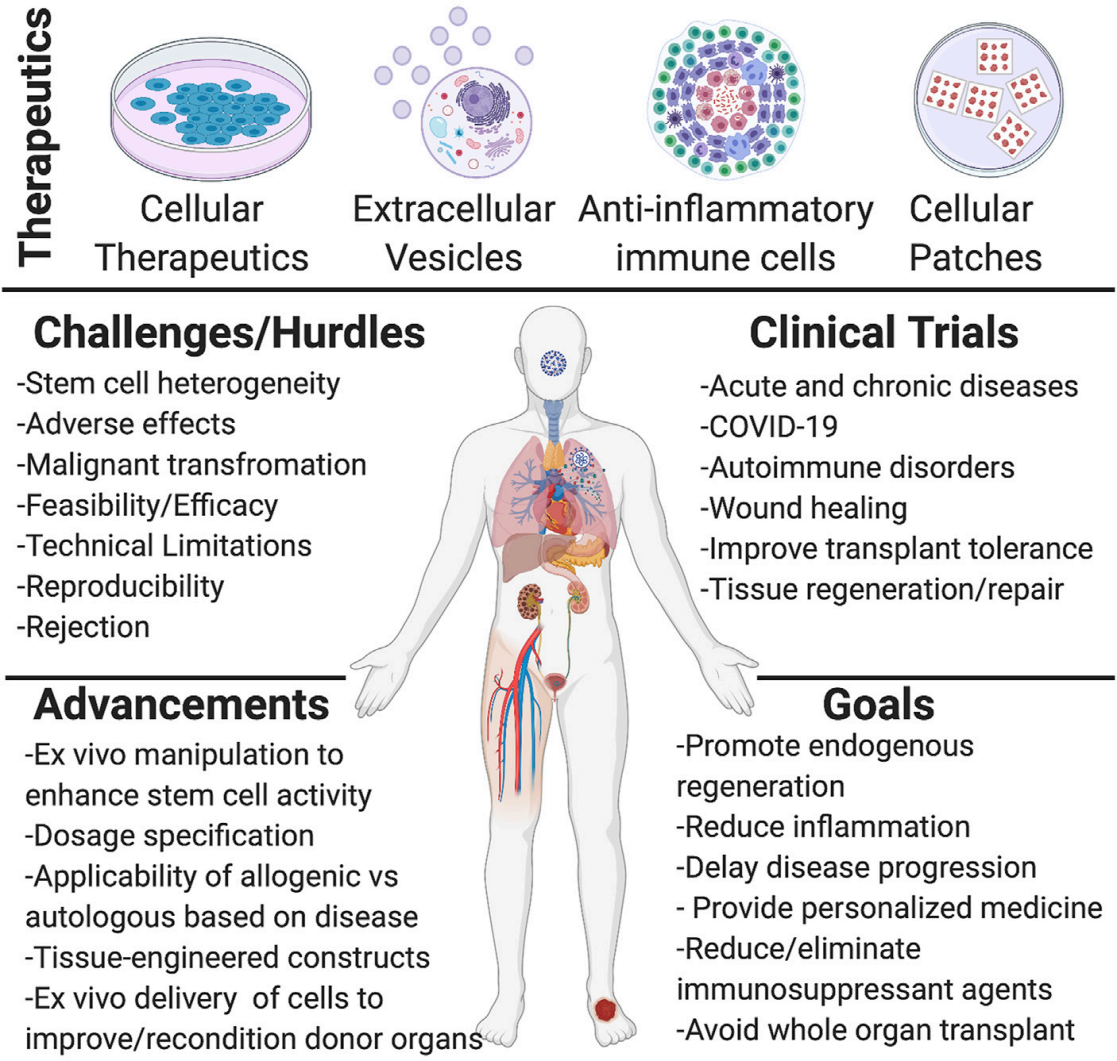
Schematic representation of regenerative medicine therapeutics and current knowledge acquired. The first half of the scheme shows the different forms of therapeutics used and investigated for regenerative medicine applications, such as different cellular materials (mesenchymal stem/stromal cells (MSCs), induced pluripotent stem cells (iPSCs), progenitor cells), extracellular vesicles (exosomes, microvesicles, and apoptotic bodies), anti-inflammatory immune cells (M2 macrophages, Treg cells, Chimeric antigen receptor transduced natural killer (CAR-NK)), and cellular patches (tissue-engineered oral mucosa graft, bilayer living cell construct, cardiac patch). The second half of the scheme shows the many challenges and hurdles currently present with cellular therapies, such as efficacy and safety and the various improvements made in regenerative medicine. A list of different targets of clinical trials is listed, along with the different overall goals from such targets. Created with BioRender.com.

Stem cell and EV therapies, along with tissue engineering, aim to deliver focused, effective patient-specific treatments that provide benefits with a single rather than lifelong intervention. Some major hurdles facing regenerative medicine is the lack of complete characterization, consistency, and standardization of cellular materials and EVs (derivation, cell/EV numbers and method of transplantation) used in clinical trials. Not all published clinical trials adhere to the International Society for Cellular Therapy (ISCT) and International Society for Extracellular Vesicles (ISEV) guidelines (Table 2). This inconsistency limits our ability to compare and contrast study outcomes and hinders the field’s progression. Although there remains challenges and uncertainties with these therapies, their potential in regenerative medicine is undeniable, and the implications of this field remain great. Improvements are continually being made to understand and utilize stem cell therapies for regenerative medicine specific to tissue type, disease, and inflammatory state along with understanding of dosage effectiveness, methods for better scalability, and guidelines for improved characterization (Figure 1). Many hurdles and limitations still exist in tissue and organ engineering, hampering researchers from the ultimate goal of whole organ generation *ex vivo*. However, the combination of novel technologies and cell-based therapies to replace, restore, or rejuvenate organ function remains appealing to investigators. The commercialization of tissue-engineered grafts for surgical use is shown to be a viable option for future clinical applications, which 1 day may provide an efficacious and innovative patient-specific therapy towards the treatment of diseases, organ loss, or damage. Thus, we can provide a more consistent and effective form of therapy by identifying and pointing out the limitations and hurdles facing regenerative medicine along with novel technologies and informative studies.

## Impact statement

Regenerative medicine aims to deliver focused, effective patient-specific treatments with lifelong benefits. Clinical trials have shown some limitations, challenges, and uncertainties with regenerative therapies; however, the field’s potential and implications remain great. Clear identification of the limitation and hurdles of regenerative medicine applications combined with the design and use of novel technologies/techniques, extracellular vesicles, and cell-based therapies to replace, restore, or rejuvenate organ function drives the future toward addressing the limitations of regenerative medicine and designing effective patient-specific treatments.
